# To Physicians

**Published:** 1856-04

**Authors:** J. A. Leonard

**Affiliations:** Whitewater, Wisconsin


TO PHYSICIANS.—I offer for sale *a Skeleton* and a set of *Anatomical Plates.* The Skeleton is prepared in the best French style, and perfectly wired, and the plates are of the size of life and accurately colored. The whole is admirably adapted either for an office or for lecturing, and will be sold cheap. Address

J. A. LEONARD, M.D.,
*Whitewater, Wisconsin.*

